# Incidence of Perforated Appendicitis during the COVID-19 Pandemic: Lessons to Be Considered in the Second Wave

**DOI:** 10.1007/s11605-021-04915-4

**Published:** 2021-02-08

**Authors:** Dörte Wichmann, Ulrich Schweizer, Daniel Wulff, Karolin Thiel, Christian Beltzer, Alfred Königsrainer, Rami Archid

**Affiliations:** 1 Department of General, Visceral and Transplantation Surgery, Tübingen University Hospital, Hoppe-Seyler-Str. 3, 72076 Tübingen, Germany; 2 Department of General, Visceral and Thoracic-Surgery, German Armed Forced Hospital Ulm, Oberer Eselsberg 40, 89081 Ulm, Germany

**Keywords:** COVID-19, Perforated acute appendicitis

## Introduction

Acute appendicitis is the most frequent disease requiring emergency surgery and has an estimated lifetime risk of 6.7 to 8.6%.^1^ Perforated appendicitis has a mortality rate of up to 5%.^2^,^3^

The COVID-19-pandemic has impacted many lives and affects healthcare systems globally. To provide sufficient capacity for SARS-CoV-2 patients, elective surgery cases have been postponed in Germany from March 16, 2020, to May 2020.

An increasing number of perforated appendicitis cases since March 16, 2020, prompted the question whether this increase can be ascribed to delayed initial medical contact after the onset of symptoms as a result of fear of contracting COVID-19 or whether there was a patient shift in our catchment area.

## Material and Methods

This is a retrospective, single-center study, approved by the local ethic committee with analysis of all patients treated for acute appendicitis in a 10-week period from March 16 to May 31 in 2018, 2019, and 2020 were considered for the study. Informed consent was obtained from all participants.

Data are available in SPSS v. 26.0.0.1 (IBM, Armonk, NY, USA) and were presented as means ± SD.

## Results

In total, 143 patients (73 male, 70 female, mean age 38.36 years) were operated for acute appendicitis. Table 1 shows the patients’ characteristics. In 2018, 12 (22.22%) of 54 patients and in 2019, 13 (30.23%) of 43 patients with acute appendicitis suffered a perforation. In 2020, during 10 weeks of the first wave of COVID-19-pandemic, 21 (44.68%) of 46 patients with acute appendicitis perforations were found (*p* = 0.039). Treatment-related characteristics are listed in Table 2.

**Table 1:** Patient characteristics according to year of treatment

	2018	2019	2020	*p* < 0.05
Total number	54	43	46	n.s.
Number of perforated appendicitis (%)	12 (22.22)	13 (30.23)	21 (45.65)	0.039
Mean age	39.63 ± 23.37	42.71 ± 21.14	38.65 ± 17.59	n.s.
Sex (number)	27 male, 27 female	20 male, 23 female	25 male, 21 female	n.s.
ASA Score				
I	37	29	26	n.s.
II	14	12	19	n.s.
III	3	2	1	n.s.

**Table 2:** Treatment characteristics of acute appendicitis according to year of treatment

	**2018**	**2019**	**2020**
Total number	54	43	46
Number of perforated appendicitis (%)	14 (25.93)	12 (27.91)	21 (45.65)
Number of standard laparoscopic appendectomies without drains (%)	37 (68.51)	30 (69.76)	30 (65.27)
Number of laparoscopic appendectomies with drains (%)	8 (14.81)	5 (11.62)	8 (17.39)
Number of laparoscopic cecal resections (%)	4 (7.41)	3 (6.98)	4 (8.69)
Number of conversions to open appendectomy (%)	1 (1.85)	3 (6.98)	0
Number of laparoscopic appendectomies with extended resections (Meckel diverticulum, ovarian cysts) (%)	2 (3.70)	2 (4.65)	3 (6.52)
Number of surgeries with primary open approach (%)	1 (1.85)	0	0
Number of redo surgeries or percutaneous drains (%)	2 (3.70)	4 (9.30)	2 (4.35)
Number of patients treated with single-dose antibiotic therapy (%)	40 (74.07)	28 (65.11)	34 (73.91)
Mean concentration of CRP at time of admission (mg/dl)	5.51 ± 6.33	6.26 ± 10.3	4.89 ± 6.53
Mean leukocyte count at time of admission (n/ml)	13,398 ± 4309	14,360 ± 3595	12,540 ± 5299
Mean delay between onset of symptoms and presentation at the emergency unit, self-reported (days)	2.23 ± 2.65	2.29 ± 1.77	1.65 ± 1.38
Mean length of hospital stay (days)	2.85 ± 3.19	3.89 ± 3.93	3.54 ± 4.58

## Discussion

Our clinical impression of seeing more complicated events of different entities, especially perforated appendicitis, was proven by this analysis. No relation between SARS-CoV-2 infections and appendicitis has been described so far.

We found a statistically significant increase in perforations during the recent period (*p* = 0.039).

Lessons to be considered in the second wave: Especially in the actual situation of increasing number of SARS-CoV-2 infections surgeons and institutions need to be prepared for advanced intraoperative findings, more intensive monitoring, more complications, and longer hospital stay in patients with acute appendicitis.
